# Metabolic patterns of sweat-extracellular vesicles during exercise and recovery states using clinical grade patches

**DOI:** 10.3389/fphys.2023.1295852

**Published:** 2023-12-07

**Authors:** Nsrein Ali, Syeda Tayyiba Rahat, Mira Mäkelä, Maryam Nasserinejad, Tommi Jaako, Matti Kinnunen, Jyrki Schroderus, Mikko Tulppo, Anni I. Nieminen, Seppo Vainio

**Affiliations:** ^1^ Laboratory of Developmental Biology, Faculty of Biochemistry and Molecular Medicine, Oulu, Finland; ^2^ Infotech Oulu, Oulu, Finland; ^3^ Flagship GeneCellNano, University of Oulu, Oulu, Finland; ^4^ Netskinmodels Cost Action CA21108, Oulu, Finland; ^5^ Research Unit of Population Health Research, Research Center, Faculty of Medicine, University of Oulu, Oulu, Finland; ^6^ Polar Electro Oy, Kempele, Finland; ^7^ Edical Research Center Oulu, Faculty of Medicine, University of Oulu, Oulu, Finland; ^8^ Resaerch Unit of Biomedicine and Internal Medicine, Faculty of Medicine, University of Oulu, Oulu, Finland; ^9^ FIMM Metabolomics Unit, Institute for Molecular Medicine Finland, University of Helsinki, Oulu, Finland; ^10^ Kvantum Institute, University of Oulu, Oulu, Finland

**Keywords:** extracellular vesicles (EVs), skin, sweat, health monitoring, metabolites, non-invasive

## Abstract

**Background:** Metabolite-based sensors are attractive and highly valued for monitoring physiological parameters during rest and/or during physical activities. Owing to their molecular composition consisting of nucleic acids, proteins, and metabolites, extracellular vesicles (EVs) have become acknowledged as a novel tool for disease diagnosis. However, the evidence for sweat related EVs delivering information of physical and recovery states remains to be addressed.

**Methods:** Taking advantage of our recently published methodology allowing the enrichment and isolation of sweat EVs from clinical patches, we investigated the metabolic load of sweat EVs in healthy participants exposed to exercise test or recovery condition. -Ten healthy volunteers (-three females and -seven males) were recruited to participate in this study. During exercise test and recovery condition, clinical patches were attached to participants’ skin, on their back. Following exercise test or recovery condition, the patches were carefully removed and proceed for sweat EVs isolation. To explore the metabolic composition of sweat EVs, a targeted global metabolomics profiling of 41 metabolites was performed.

**Results:** Our results identified seventeen metabolites in sweat EVs. These are associated with amino acids, glutamate, glutathione, fatty acids, creatine, and glycolysis pathways. Furthermore, when comparing the metabolites’ levels in sweat EVs isolated during exercise to the metabolite levels in sweat EVs collected after recovery, our findings revealed a distinct metabolic profiling of sweat EVs. Furthermore, the level of these metabolites, mainly myristate, may reflect an inverse correlation with blood glucose, heart rate, and respiratory rate levels.

**Conclusion:** Our data demonstrated that sweat EVs can be purified using routinely used clinical patches during physical activity, setting the foundations for larger-scale clinical cohort work. Furthermore, the metabolites identified in sweat EVs also offer a realistic means to identify relevant sport performance biomarkers. This study thus provides proof-of-concept towards a novel methodology that will focus on the use of sweat EVs and their metabolic composition as a non-invasive approach for developing the next-generation of sport wearable sensors.

## Introduction

Currently, wearable devices including sport watches, smart rings and others are widely valued for monitoring wellbeing and promoting physical activity, as well as social interaction (Kim et al., 2019; Promphet et al., 2021). Given that most of these sensors rely on electrical signal generated by temperature or muscle pulsation like the heart, recording different parameters during physical activities is an open challenge. To overcome these challenges, we and others reported that sweat - one of several natural body fluids, such as saliva, urine, and tears - may offer avenues for non-invasive testing of a variety of biomarkers and their association with health parameters. Furthermore, a particular attention was given to specific analytes of sweat when developing new generation of wearables, commonly lactate, pyruvate, urea, and certain ions (Gao et al., 2016; Ghaffari et al., 2021; Ibrahim et al., 2022; Wang et al., 2022; Gao et al., 2023; Yang et al., 2023).

Sweat is excreted by sweat glands, known as one of the skin appendages, which differ in their function, distribution, and cellular structure (Baker, 2019; Chen et al., 2020), however, whether sweat glands’ responses against certain challenges may influence the concentration of analytes in sweat remains an open question. The new discovery of extracellular vesicles (EVs) as unique signaling messengers composed of diverse array of molecular components biomolecules (Boilard, 2018; van Niel et al., 2018; Mathieu et al., 2019), in which their metabolic profiling can be affected by cellular responses towards a variety of stressors, may represent valuable tools for exploring the question addressed above.

Extracellular vesicles are categorized according to their cellular origin and size. Exosomes are 10–150 nm in diameter and are assembled in the multivesicular bodies (MVBs). The macrovesicles are 500–1,000 nm in diameter and are derived directly from the donor cell plasma membrane. Both EV types are released into the extracellular space and transported throughout the body via bodily fluids (van Niel et al., 2018; Doyle and Wang, 2019). Owing to these distinct properties, EVs were widely studied and numerous reports suggest that EVs may offer the foundation for identifying the molecular patterns of frequently occurring diseases (Piombino et al., 2021; Testa et al., 2021; Zhao et al., 2021). Moreover, considering that sweat contains a variety of vesicles, a particular attention was given to explore the molecular composition of EVs presented in sweat, with the aim of developing non-invasive diagnostic strategies (Yu et al., 2017; Cheng and Hill, 2022; Yang et al., 2023). Our recent publication demonstrating, for the first time, that metabolite levels from sweat EVs may serve as a means for reflecting metabolic changes in healthy and type 2 diabetes groups after heat exposure, is highly relevant. Furthermore, with the aid of clinical patches, we described a new non-invasive methodology allowing the cost-effective enrichment and the isolation of sweat EVs from healthy and diseased participants (Rahat et al., 2023).

In this current study, we employed our recently published methodology (Rahat et al., 2023) for assessing the metabolic signatures of sweat EVs in response to exercise and after recovery. Healthy participants were recruited to take part in this study. Clinical patches were attached to participants’ skin, on their back during exercise test (25–30 min) or under recovery conditions (30 min). Subsequently, sweat EVs were isolated from the clinical patches and further characterized using nanoparticle tracking analysis (NTA), electron microscopy, and Western blot. A targeted metabolomic analysis of the sweat patch enriched EVs revealed 17 metabolites which were determined by chromatography analysis. Furthermore, when comparing the metabolite levels in sweat EVs isolated from healthy participants during exercise tests, with their levels in sweat EVs isolated from patches obtained after recovery, our finding indicate that physical activity resulted in a significant upregulation of the production of few metabolites belonging to different biological and molecular pathways. Statistical analysis of the data revealed that the level of metabolites, mainly myristate, in sweat EVs may reflect a correlation with blood glucose, heart rate, and respiratory rate levels.

Overall, this study is the first to demonstrate the enrichment of sweat EVs from clinical patches during exercise tests and the quantification of their metabolites, therefore, lays the groundwork for large-scale population studies for monitoring sport performance and recovery programs, in particular for athletes.

## Material and methods

### Study design

The controlled study has been approved by the Northern Ostrobothnia Hospital District Ethics committee (EETMK:5/2022). The inclusion criteria for this study were: BMI 15–25, age 18–50 years, fasting glucose blood between 3.9–5.6 mmol/L, and HbA1c below 42 mmol/mol (5.7%–6%). The exclusion criteria for this study were: history of any cardiovascular diseases, blood pressure of >160 mmHg systolic/and >100 mmHg diastolic, - medications that may affect blood pressure.

Prior to the experiment, all individuals were provided a participation information document that contained all details about the research proposal, procedures, aims, risks, and contact details of the research team. Upon acceptance, participants were invited to read and sign the consent document. In this study, all participants declared no conflict of interest.

All volunteers who met the inclusion and exclusion criteria were asked to meet researchers on two occasions: the first visit at Oulu University and the second visit at Polar Electro Oy, Kempele, Finland.

During the first visit, volunteers were invited to read and sign the informed consent document. Additionally, weight, height, body fat, fasting blood glucose, and arterial blood pressure were measured. Standard lead-II electrocardiography (Cardiolife, Nihon Kohden, Tokyo, Japan), breathing frequency (Respiratory Belt Transducer, ADInstrument, Australia), and blood pressure by finger plethysmography (Nexfin, BMEYE Medical Systems, Amsterdam, Netherlands) were recorded at 5min intervals in a seated position with a sampling frequency of 1,000 Hz (PowerLab 8/35, ADInstruments). Mean heart rate, root mean square of successive differences in R-R intervals (the time elapsed between two successive R-waves of the QRS signal on the electrocardiogram) (rMSSD, ms), spectral power densities (fast Fourier transform, length 512 beats) at low-frequency (LF, 0.04–0.15 Hz, ms2) and high-frequency (HF, 0.15–0.40 Hz, ms2) components of heart rate variability (HRV), and their ratio LF/HF were analyzed. For the baroreflex sensitivity (BRS) analysis, a fast Fourier transform (Welch method, segments of 128 samples with 50% overlap, length 1,024 samples) was performed to analyze the LF power of the R-R interval and systolic blood pressure (SBP) oscillations (LF ms2, LFSBP mm Hg2) for subsequent analysis of BRS by the alpha, if sufficient coherence (≥0.5) between LF oscillations in the R-R interval and SBP was verified (Kiviniemi et al., 2014). To note, only nine - three women and six men - out of ten healthy participants were involved in the ECG measurements and the obtained data was then used for conducting correlation analysis with resting heart rate and resting blood pressure levels.

### Exercise test in the laboratory

The experiment was performed at the research center at Polar Electro Oy, Kempele, Finland, where -ten healthy volunteers (three women and seven men) were exposed to exercise test followed by a recovery period. All experiments were performed at room temperature (+22C). All volunteers wore light (sport) clothing (shorts, t-shirt, and socks). During the first 30 min, in a seated position, clinical patches were then attached to participants’ skin, on their back. Following 3 minutes of sitting on the saddle, volunteers were invited to start cycling. The experiment started with an intensity of 40 W for everyone. Resistance increases were 15 W for females and 20 W for males and were increased every 2 minutes. To note, the duration of exercise test was different for each participant as showed by the average for females and males (Table 1). Cycling was stopped when the subject could not continue pedaling or wanted to stop (exhaustion). A cool down period consisting of 40 W resistance cycling during 5 minutes was applied followed by a sitting period of 3 minutes. Subsequently, patches were removed, and the total sweat absorbed was obtained via weighing the patches from each participant before and after the exercise test. Finally, right after exercise, all volunteers were invited to rest, in a seated position, for 30 min, referred to as the recovery condition, where new skin patches were attached to participants’ skin, on their back, then collected at the end of this period.

**TABLE 1:** Characteristics of the participants in this study. Data are means ± SD. For exercise and recovery studies, ten healthy participants (N = 10; three women and seven men) were included and characterization of their age, height, weight, blood glucose, BMI, and exercise were presented. For ECG measurements, only nine healthy participants, three women and six men, were involved. N. Number.

Variable	Healthy participants	
Gender	Women	Men
Number	3	7
Age	37.7 ± 6.6	38.7.± 3.6
Height. cm	167 ± 3.7	175.9 ± 6.0
Weight. Kg	63.07 ± 9.4	76.7 ± 13.1
Blood glucose (mmol/L)	5.3 ± 0.2	5.3 ± 0.3
Body mass index (BMI. Kg/m2)	18.9 ± 2.8	21.2 ± 2.0
Exercise Duration (minutes)	25.0 ± 1.6	29.4 ± 5.5
Exercise maximal Intensity (Watts)	180.0 ± 16.3	262.9 ± 61.4
Number	3	6
Resting blood pressure (mmHg)	114.0 ± 5.1	114.0 ± 6.4
Systolic	74.7 ± 10.0	72.5 ± 7.7
Diastolic		
Resting heart rate	63.3 ± 4.3	54.8 ± 11.5

Samples collected from ten of the healthy participants were further used for sweat EVs isolation followed by a targeted metabolomics analysis.

### Isolation of sweat EVs

The EVs from sweat were isolated as previously described (Rahat et al., 2023). After sweat collection, the Sorbact^®^ dressing was opened, and the superabsorbent layer was dissolved in 40 mL (1x) PBS, before being filtered through a 40 mm sieve, to remove large particles and pieces of fiber. The filtrate was then re-filtered through 0.8 µm vacuum filter units (Thermo Fisher Scientific). The filtrate was centrifuged in a Sorvall AH-629 rotor at 100,000 g for 3 h in a Sorvall Ultracentrifuge Machine WX ultra 90 (VWR, Thermo Electron Corporation). The supernatant was collected, and the pellets were washed twice using 1x PBS followed by centrifugation at 100.000 *g* for 2 h, and finally resuspended in 40–60 µL of 1x PBS.

### Targeted LC-MS metabolomics analysis

Metabolites were extracted from sweat EVs and their intensity was measured using a targeted LC-MS metabolomics approach, as described in Rahat et al., 2023 (Rahat et al., 2023). The metabolite intensity levels were normalized to sample volume and sweat EVs concentration.

### Western blot

The quantity of protein in the EV samples was calculated using BCA assay (Pierce™ BCA Protein Assay Kit) according to the manufacturer’s recommendations. Equal amounts of total protein (10 µg) were separated by SDS-PAGE and electrophoretically transferred to nitrocellulose membranes (741,280, BioTop, Germany). The membranes were then incubated overnight at 4 °C with the following primary antibodies (anti-HSP70 [sc-373867], and anti-CD63 [sc-5275]) from Santa Cruz Biotechnology and then with the secondary antibody (P0260, Aligent Tech, Glostrup, Denmark). The blots were then developed using Pierce™ ECL plus Western blotting substrate (32,132, Thermo Fisher Scientific, United States).

### NanoSight - Nanoparticle tracking analysis

Nanoparticle tracking analysis (NTA) was performed using a NanoSight NS300 (NanoSight Ltd, Amesbury, UK) equipped with a 405 nm laser. At least four 60 s videos were recorded for each sample, with camera level 14 and detection threshold set at 5. Temperature was monitored throughout the measurements. Videos recorded for each sample were analyzed with NTA software version 3 to determine the concentration and size of measured particles with the corresponding standard error. For analysis, auto settings were used for blur, minimum track length and minimum expected particle size. Double-distilled H_2_O was used to dilute the sample.

NTA technique was used for determining the concentration. EVs were resuspended in 1x PBS following ultracentrifugation. Only 1–2 µL of EVs suspension is used to perform NTA. The EVs are diluted to 1:300, 1:400, or 1:500 in distilled sterile water. Initial trials involved more concentrated dilutions, with adjustments made to maintain particle concentrations within the desired range of 5.5 × 10^7 to 9.0 × 10^8 particles per ml. The 1:300 dilution proved consistently efficacious for us. The diluted sample volume typically ranged from 600 to 1,000 µL.

### Electron microscopy_ negative staining

Isolated EVs were settled on Formvar carbonated copper grid (glow-discharged). Following fixation with 1% glutaraldehyde, the grid was washed with distilled water and stained with neutral 2% UA (Uranyl acetate). The grid was subsequently coated with 2% methylcellulose-UA solution and, following 10 min incubation, the excess fluid was carefully removed, and grids were air dried. EVs were visualized using a Tecnai Spirit G2 transmission electron microscope and images were taken with a Veleta CCD camera and Item software (Olympus Soft Imaging Solutions GMBH, Munster, Germany).

### Statistical analysis

To assess the association between the concentration of sweat EVs isolated from patches during exercise and the following parameters, such as fasting blood glucose, BMI, resting heart rate, resting blood pressure, and resting respiration frequency, a Spearman’s rank correlation was applied. All analyses were performed with R software version 4.2.0. GraphPad Prism software, version 7, was used for statistical analyses. The two-tailed Student t-test was employed and *p-values less than 0.05 were considered significant.

## Results

### Characterization of sweat EVs enriched in clinical patches during the exercise test

We recently reported, for the first time, a new methodology for isolating and characterizing EVs from sweat collected in clinical patches, following heat exposure, under resting conditions. Using the same protocol, we aimed to examine the presence of EVs in sweat-enriched patches during exercise tests and to investigate their metabolic signatures. For this purpose, we recruited twenty healthy subjects to take part in this study. The selection criteria of the participants were: age, sex, blood glucose, and BMI (Table 1).

**FIGURE 1:**
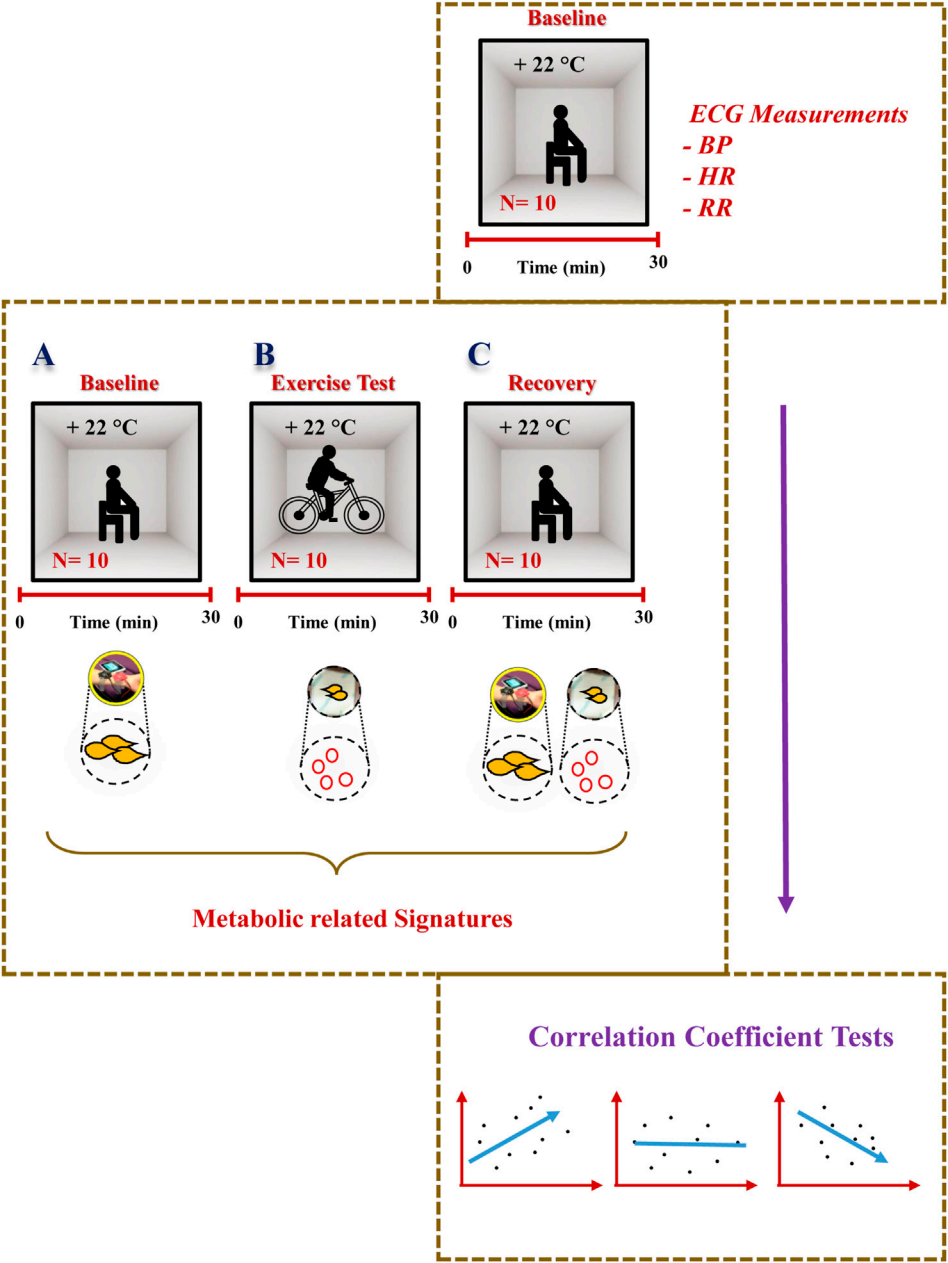
Experimental design. Detailed description of the study design and the sweat EVs isolation and characterization steps. PBS: phosphate buffered saline. min: minutes. hrs: hours.

The experimental design is described in Figure 1. Prior to the exercise tests, prepared patches were attached to participants’ skin, on their back, and then participants were invited to start cycling. Following the exercise test, the patches were carefully removed and stored for further investigation.

To explore the presence of EVs in the sweat-enriched patches, a series of filtrations was employed as previously described (Rahat et al., 2023). Following isolation, sweat EVs were then characterized using Nano-particle Tracking Assay (NTA), electron microscopy (EM), and Western blot methodologies.

**FIGURE 2:**
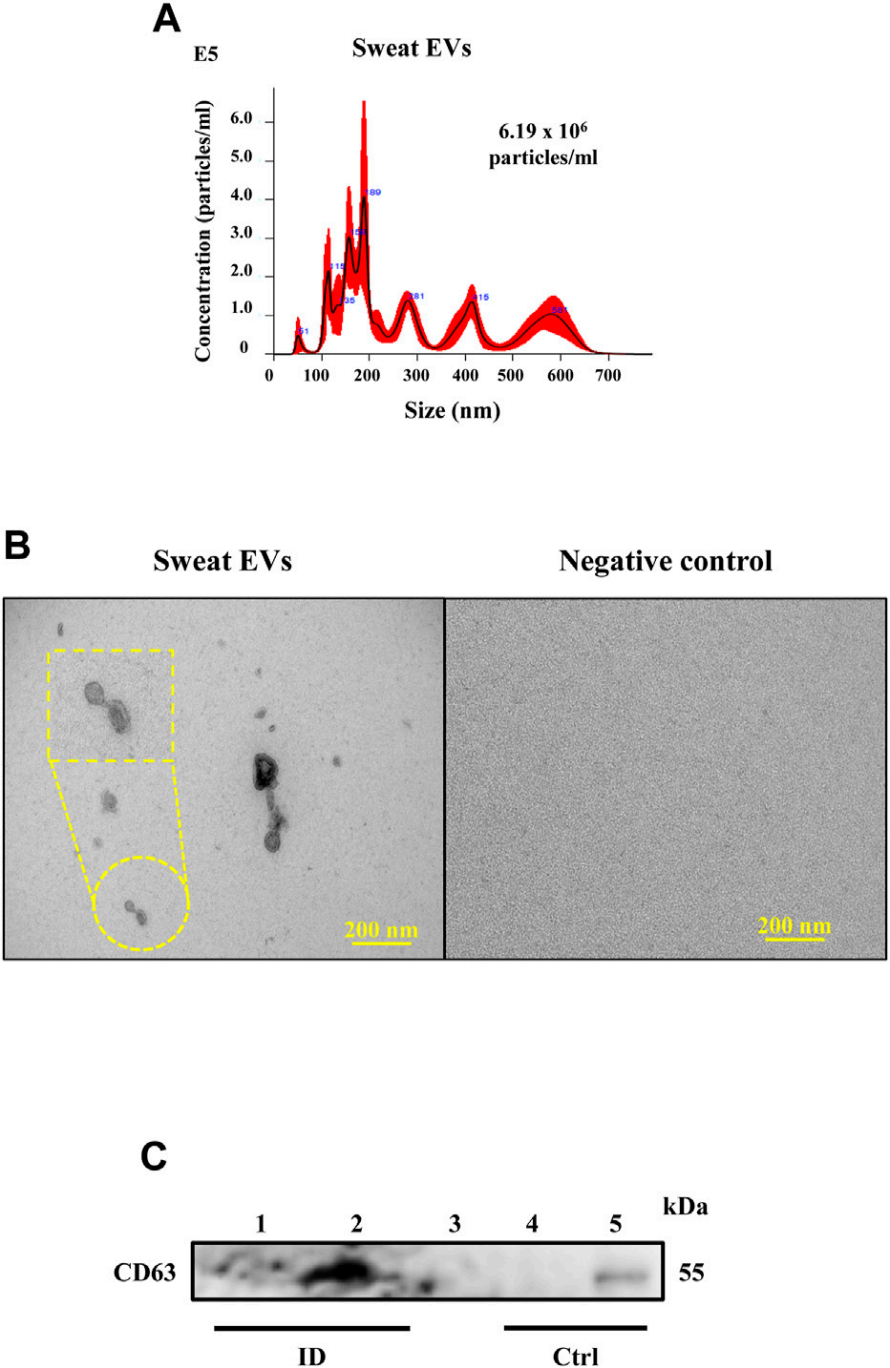
Characterization of sweat EVs. **(A)**. NTA analysis of sweat EVs isolated from healthy participants during the exercise test. **(B)**. Electron microscopy analysis. Negative staining of sweat EVs isolated from patches, control refers to the negative patch (has not been attached to the patient) but processed in the same way as the positive patches. Scale bar = 200 nm. **(C)**. Western blot analysis of CD63 in sweat EVs isolated from two healthy participants during exercise test (lanes 1 and 2). Control lanes (Ctrl) refer to: 1- negative controls (lane 3: 1x PBS and lane 4: non-enriched sweat patch, that has not been attached to a participant, and 2- positive control that refer to EVs extracted from human keratinocytes (lane 5).

The NTA data revealed that sweat EVs were 100–700 nm in diameter, with EVs that were 100–250 nm in diameter being the most predominant (Figure 2A). NTA analysis also enabled the estimation of the overall quantity of enriched EVs in the patches from the different participants, with an average number being 2.94×10^10^ ± 2.9×10^10^ particles/mL (Supplementary Table S1).

In line with the NTA data, subjecting sweat EVs to EM based analysis confirmed that the EVs differed in size (Figure 2B). Given our recent report (Rahat et al., 2023) demonstrating that the sweat EVs dominantly express the CD63 when compared to other EVs markers such as CD9 and CD81, we performed Western blot analysis to evaluate the CD63 expression levels. Our finding using sweat EVs samples isolated from two subjects showed the presence of a band at 54 kDa, which corresponded to CD63 (lanes 1 and 2) and confirmed using a positive control (lane 5: EVs extracted from human keratinocytes). However, no band was observed in control samples (lane 3: 1x PBS; lane 4: non-enriched sweat patch, which had not been attached to a participant, the contents of which were extracted and isolated following the same protocol of the sweat-enriched patches) (Figure 2C)

To conclude, our study demonstrates the successful enrichment and isolation of EVs from sweat collected with the aid of clinically approved skin patches during exercise tests, thus substantiating our previously reported methodology.

### Metabolic composition of extracellular vesicles from sweat during exercise tests

Given our recent report, demonstrating that sweat EVs isolated from healthy participants after exposure to heat produce particular “cargo” metabolites (Rahat et al., 2023), we aimed to characterise the metabolic constitution of sweat EVs collected from healthy participants during an exercise test. To address this, a targeted metabolomics profiling of 41 metabolites we conducted.

Of the 41 metabolites analyzed, only 17 metabolites were identifiable using a chromatography approach (Supplementary Table S2). On closer examination of the values of metabolite abundances in sweat EVs, an important difference in their intensity levels between healthy individuals was noted (heatmap, Supplementary Figure S1).

To quantitively compare the differences in the metabolite intensities of sweat EVs between healthy individuals during exercise, the metabolite peak area fold changes were calculated. Based on this, and the relation of each metabolite to well defined metabolic signaling profiles, the identified metabolites were subsequently categorized into subgroups, such as those related to amino acids, glutathione, glutamate, fatty acids, glycolysis, and creatine metabolism pathways.

**FIGURE 3:**
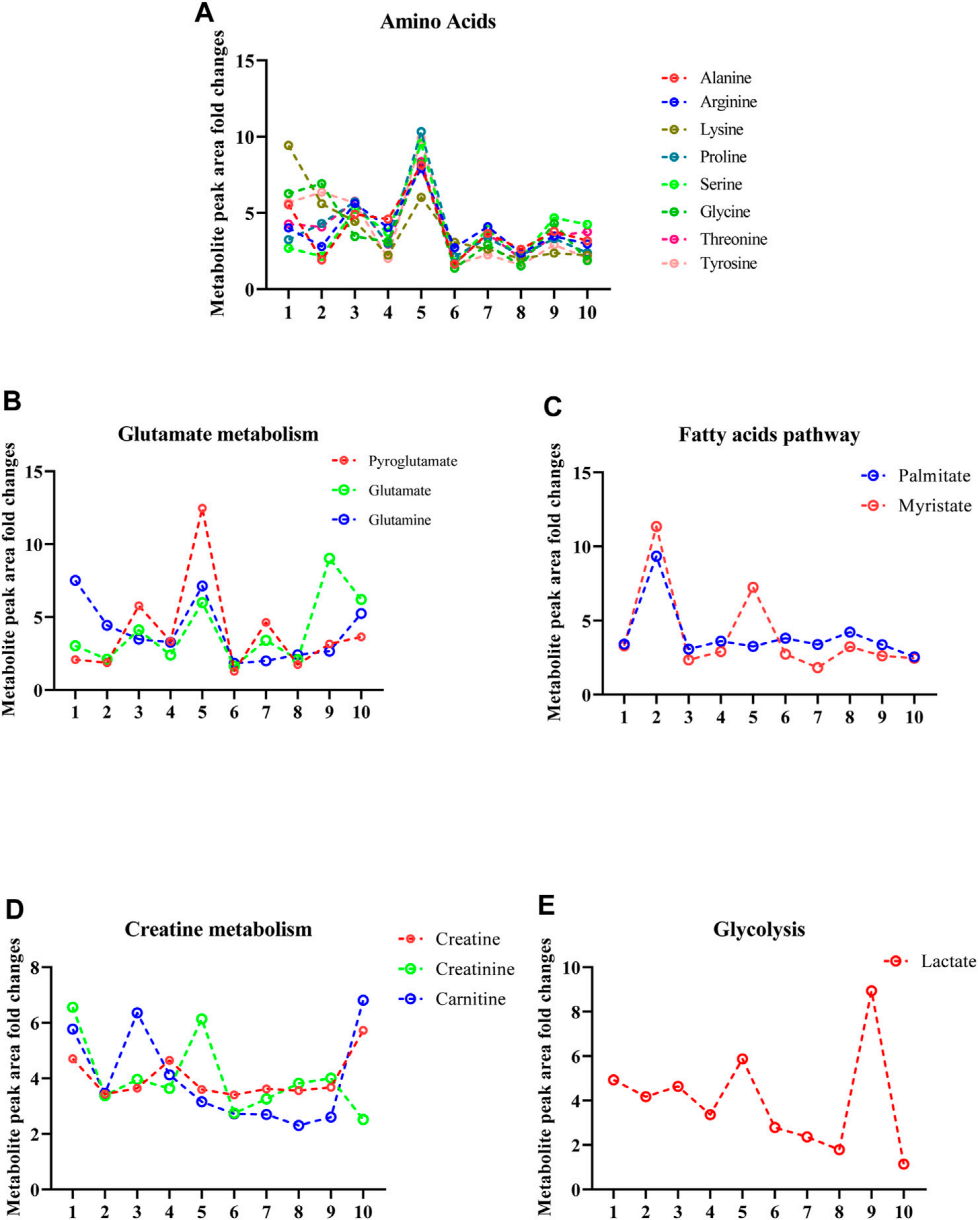
Sweat EVs exhibit specific metabolic qualities. **(A)**. Metabolite signatures of the amino acid pathway including (alanine, arginine, glycine, lysine, proline, serine, threonine, and tyrosine). **(B)**. Metabolite signatures of glutathione and glutamate pathways. The metabolites are pyroglutamate, glutamate, and glutamine. **(C)**. Metabolite signatures of the fatty acid pathway. The metabolites are palmitate and myristate. **(D)**. Metabolite signatures of the creatine pathway. The metabolites are creatine, creatinine, and carnitine. **(E)**. Metabolite signatures of glycolysis pathway including lactate. Data are metabolite peak area fold changes ±SEM after normalization to the total EVs concentrations (particles/mL) and to the negative control (patch without sweat sample). N = 10; three women and seven men.

Metabolites from the amino acid metabolism pathway contained alanine, arginine, lysine, proline, serine glycine, threonine, and tyrosine (Figure 3A). Those metabolites identified from glutathione and glutamate metabolism pathways included pyroglutamate, glutamate, and glutamine (Figure 3B), while those arising from the fatty acid pathway included myristate, and palmitate (Figure 3C). Metabolites from the creatine pathway included creatine, creatinine, and carnitine (Figure 3D). Finally, a notable component from the glycolysis pathway was lactate, which is a well-established metabolite for monitoring physical activity during sport (Figure 3E).

### Metabolic profiling of sweat EVs during exercise test in comparison to at recovery condition

To investigate whether exercise may influence the metabolic patterns of sweat EVs collected from healthy participants in comparison with samples collected during a rest period of 30 min and referred to as a recovery condition, the 17 metabolites previously identified (Figure 3), were analyzed from the clinical patches collected from healthy individuals following recovery condition.

Via performing NTA analysis, our findings revealed no difference in the concentration of sweat EVs isolated from healthy participants at recovery condition, with an average number being 2.69×10^10^ ± 2.25×10^10^ particles/mL, when compared with the average concentration that was observed in sweat EVs isolated during exercise tests (Supplementary Table S1). Moreover, no differences in the size of the sweat EVs isolated at recovery condition when compared to these isolated during exercise tests.

Of the 17 analyzed metabolites, only 16 metabolites were identified in sweat EVs collected from healthy participants during recovery from exercise. Closer analysis of the raw data obtained using chromatography revealed that lysine was not detected in any of the ten sweat EVs samples collected from healthy participants in the recovery condition, however, this metabolite was identified in sweat EVs collected during the exercise test (Supplementary Table S2).

We next aimed to quantitively compare the differences in the metabolite levels of sweat EVs collected during the exercise test with those collected following recovery conditions. To this end, the metabolite intensity (peak area) fold changes of sweat EVs collected from healthy participants during exercise test in reference to their levels in sweat EVs collected following the recovery condition, were calculated.

**FIGURE 4:**
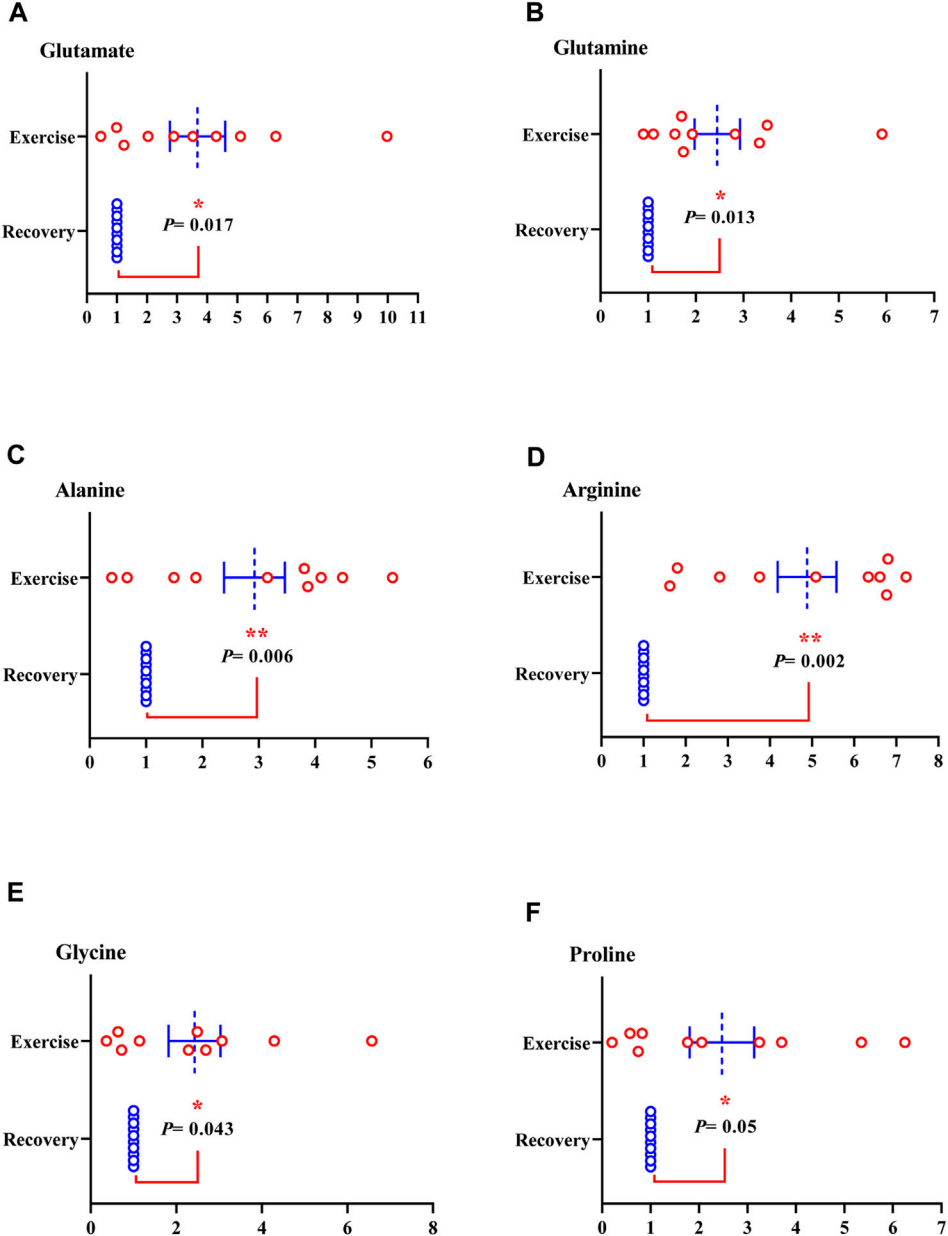
Metabolites from sweat EVs may mirror metabolic variances during the exercise test when compared to recovery state in healthy individuals. Metabolite levels of glutamate **(A)**, glutamine **(B)**, alanine **(C)**, arginine **(D)**, glycine **(E)**, and proline **(F)**. Data are metabolite peak area fold changes ±SEM after normalization to the total EVs concentrations and to the negative control. Two-tailed Student’s t-test was used in A (*p = 0.017), in B (*p = 0.013), in C (**p = 0.006), in D (**p = 0.002), in E (*p = 0.043), and in F (*p = 0.05). N = 10; three women and seven men.

**FIGURE 5:**
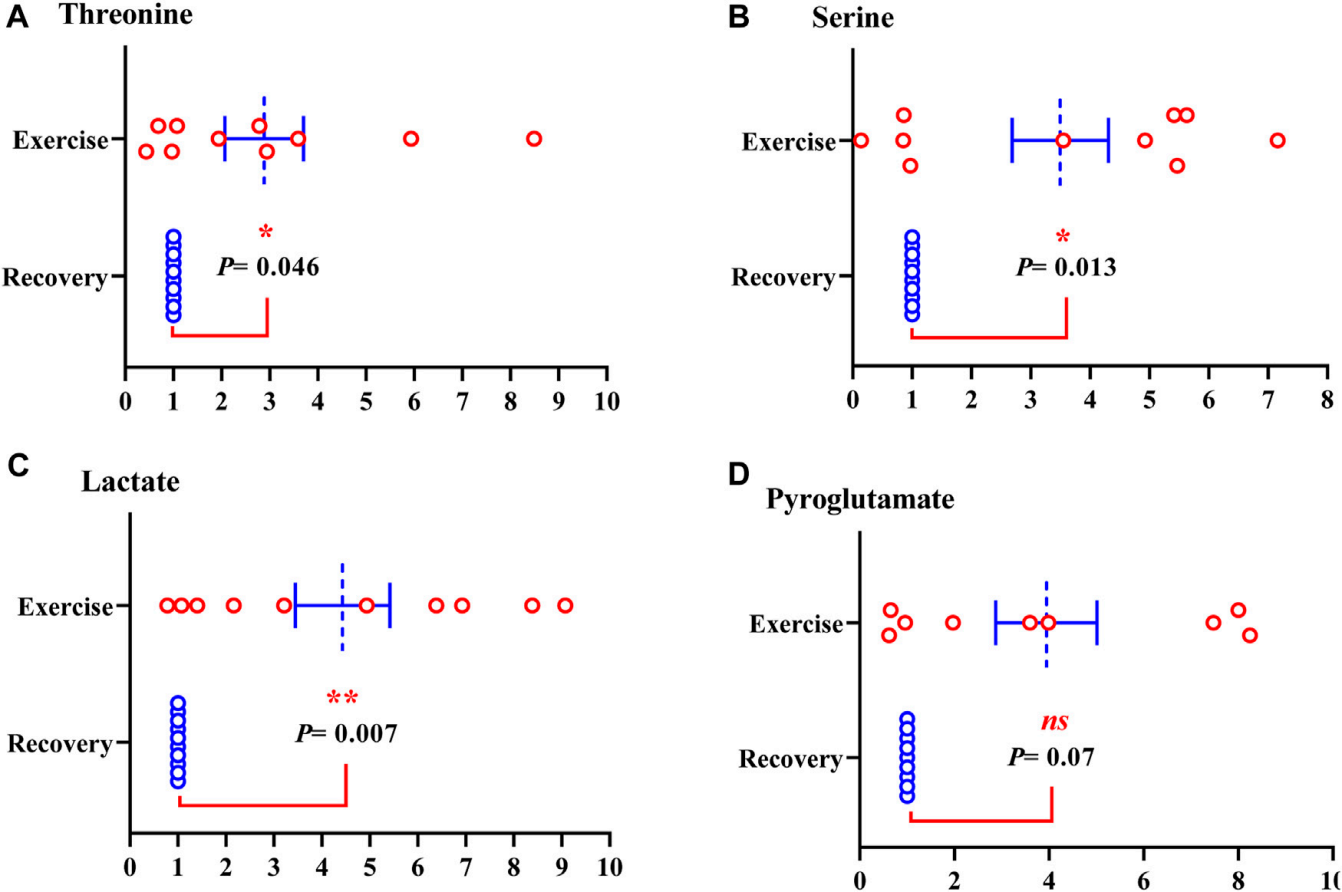
Metabolites from sweat EVs may mirror metabolic variances during the exercise test when compared to recovery state in healthy individuals. Metabolite levels of threonine **(A)**, serine **(B)**, lactate **(C)**, and pyroglutamate **(D)**. Data are metabolite peak area fold changes ±SEM after normalization to the total EVs concentrations and to the negative control. Two-tailed Student’s t-test was used in A (**p = 0.046), in B (*p = 0.013), and in C (**p = 0.002), in C (ns. not significant, *p* = 0.07). N = 10; three women and seven men.

Of the metabolites analyzed, the level of nine metabolites was significantly increased in sweat EVs isolated from healthy participants during the exercise test compared to their levels in sweat EVs isolated following the recovery condition. The metabolites were glutamate, glutamine (glutamate metabolism pathway) (Figures 4A, B); alanine, arginine, glycine, proline, threonine, and serine (amino acid pathway) (Figures 4C–E; Figures 5A,B); and lactate (glycolysis pathway) (Figure 5C).

Furthermore, the intensity of pyroglutamate (glutathione pathway), tyrosine (amino acid pathway), carnitine and creatinine (creatine pathway) were elevated, although not statistically significant, in sweat EVs isolated from healthy participants during exercise test compared to their levels in sweat EVs isolated following recovery condition (Figure 5D; Supplementary Figure S1B, C: Supplementary Figure S2A-C; respectively).

No changes were observed in the metabolite levels of creatine (creatine metabolism), and palmitate (fatty acids pathway) (Supplementary Figure S2B, C). However, a decrease of myristate was observed (fatty acid pathway) in sweat EVs isolated from healthy participants during the exercise test compared with their levels in sweat EVs isolated following the recovery condition (Supplementary Figure S2D).

Our data illustrate that the metabolic profiling of sweat EVs may provide a useful means for recording the individualized recovery responses following a physical activity and hence may facilitate the development of protocols for monitoring sport performance for athletes. However, a larger number of participants would be required for further validation.

### Association of the metabolite levels in sweat EVs with blood glucose and BMI

To assess a possible correlation between blood glucose and the levels of metabolites in sweat EVs that had accumulated into the skin patches during the exercise test, a Spearman’s rank association was performed.

Our data established that the levels of myristate (fatty acid pathway) showed a significant indirect correlation with blood glucose levels. While no evidence of association between the remaining metabolite levels and blood glucose levels was observed (Table 2).

**TABLE 2:** Spearman’s rank association between blood glucose and the metabolite levels in the sweat EVs isolated from healthy participants (N = 10; three women and seven men) during exercise. *p < 0.05 is significant indicated as bold numbers.

Variable	Correlation with blood glucose	*P.* Value
Pyroglutamate	0.30	0.42
Creatine	−0.30	0.42
Creatinine	−0.36	0.34
Glycine	−0.18	0.76
Alanine	0.27	0.48
Arginine	−0.20	0.59
Carnitine	−0.11	0.78
Glutamate	0.20	0.50
Glutamine	−0.47	0.2
Lactate	0.07	0.86
Proline	0.37	0.32
Serine	0.35	0.35
Threonine	0.21	0.58
Tyrosine	−0.02	0.96
Myristate	−0.71	0.03 *
Palmitate	−0.48	0.19

When investigating the correlation between the level of metabolites in sweat EVs during the exercise test and BMI, our results revealed no relationship between these two variables. However, glutamate levels showed a trend of inverse association with BMI, which was supported by the *p*-value (Table 3).

**TABLE 3:** Spearman’s rank correlation between BMI and the metabolite levels in the sweat EVs isolated from healthy participants (N = 10; three women and seven men) during the exercise test. BMI. Body mass index.

Variable	Correlation with BMI	*P.* Value
Pyroglutamate	−0.48	0.19
Creatine	−0.08	0.84
Creatinine	−0.33	0.38
Glycine	−0.44	0.23
Alanine	−0.3	0.43
Arginine	−0.27	0.48
Carnitine	0.25	0.52
Glutamate	−0.61	0.08
Glutamine	−0.07	0.86
Lactate	−0.47	0.19
Proline	−0.35	0.36
Serine	−0.58	0.10
Threonine	−0.44	0.24
Tyrosine	−0.13	0.74
Myristate	−0.1	0.81
Palmitate	0.35	0.36

Together, our results reveal a trend of correlation between few metabolic markers in sweat EVs during exercise and blood glucose levels but not with BMI. However, further investigations are required to confirm this observation.

### Changes in the metabolites contained in sweat EVs in relation to cardiovascular system function

**S**ystematic physical activity and exercise are known to impact health and wellbeing, leading to an increase in life expectancy. This might be explained through the negative association between sport or physical activity and heart rate (HR), commonly referred to as resting heart rate (RHR) (Reimers et al., 2018). In addition, an inverse association between exercise or physical activity and blood pressure levels was reported (Schweiger et al., 2021). Consequently, we questioned whether the concentration of metabolites in sweat EVs isolated from healthy participants during exercise may be linked to cardiovascular system functions while resting. To address these relationships, Spearman’s rank association was employed. To note, the different correlations were conducted using data collected from nine healthy participants.

### Resting heart rate levels

We first challenge the association between the level of metabolites in sweat EVs during the exercise test and resting heart rate levels. Our findings showed that the level of pyroglutamate and serine metabolites (glutathione and serine pathways) were significantly directly associated with resting heart rate levels. Judged by the *p*-value, a trend of direct relationship between the level of alanine metabolite (amino acids) with resting heart rate levels was demonstrated (Table 4). Moreover, the level of myristate and palmitate metabolites (fatty acid pathway) showed an indirect relationship with resting heart rate levels, which was significant (Table 4).

**TABLE 4:** Spearman’s rank correlation between resting heart rate and the metabolite levels in sweat EVs isolated from healthy participants (N = 9; three women and six men) during exercise. *p < 0.05 is significant indicated as bold numbers.

Variable	Correlation with resting heart rate	*P.* Value
Pyroglutamate	0.73	0.031 *
Creatine	0.28	0.46
Creatinine	0.1	0.81
Glycine	0.35	0.35
Alanine	0.633	0.07
Arginine	−0.22	0.57
Carnitine	−0.05	0.91
Glutamate	0.46	0.20
Glutamine	−0.31	0.41
Lactate	0.16	0.68
Proline	0.42	0.27
Serine	0.75	0.02 *
Threonine	0.34	0.36
Tyrosine	−0.05	0.91
Myristate	−0.76	0.02 *
Palmitate	−0.81	0.01 *

### Resting mean blood pressure levels

We next examined the association between the metabolic composition of sweat EVs isolated from nine healthy participants, during the exercise test, and resting mean blood pressure levels. Our data highlighted that the level of glutamate showed a trend of direct correlation with resting mean blood pressure levels (Table 5). While the level of myristate (fatty acid pathway) showed a trend of inverse relationship with resting mean blood pressure levels (Table 5).

**TABLE 5:** Spearman’s rank correlation between resting mean blood pressure and the metabolite levels in the sweat EVs isolated from healthy participants (N = 9; three women and six men) during exercise.

Variable	Correlation with resting mean blood pressure	*P.* Value
Pyroglutamate	0.33	0.39
Creatine	0.55	0.12
Creatinine	0.09	0.81
Glycine	−0.11	0.77
Alanine	0.31	0.42
Arginine	−0.09	0.81
Carnitine	0.16	0.68
Glutamate	0.60	0.08
Glutamine	−0.35	0.35
Lactate	0.48	0.18
Proline	0.27	0.48
Serine	0.42	0.26
Threonine	−0.12	0.75
Tyrosine	0.18	0.65
Myristate	−0.62	0.07
Palmitate	−0.48	0.19

To gain insight into the relationship between metabolite levels in sweat EVs isolated from nine healthy participants during exercise and the different measures related to heart function such as systolic blood pressure, diastolic blood pressure, and low frequency of systolic blood pressure levels, a Spearman’s rank test was performed.

Our data indicated that the level of the majority of metabolites in sweat EVs isolated from healthy participants, during exercise, showed no relationship with systolic, diastolic, and low frequency resting blood pressure (Supplementary Table S3-5).

In line to its correlation with the mean blood pressure levels, importantly, the level of metabolite myristate was the only to demonstrate a significant inverse correlation with systolic, diastolic, and low frequency resting blood pressure (Supplementary Table S3-5).

### Resting respiration rate levels

Respiration rate is an essential physiological parameter for health monitoring and can be indirectly measured by electrocardiogram. Any sign of altered respiratory rate may serve as an early warning in disease diagnosis (Cretikos et al., 2008; Fleming et al., 2011). Given this, we addressed the relationship between the levels of resting respiration and the level of metabolites in sweat EVs isolated from nine healthy participants during exercise.

Of the 16 metabolites analyzed, the level of the glutamate metabolite in sweat EVs isolated from healthy participants showed a negative correlation with resting respiration rate levels, which was statistically significant (Table 6). While the myristate and palmitate metabolites demonstrated a significant positive correlation with resting respiration rate levels (Table 6).

**TABLE 6:** Spearman’s rank correlation between resting respiration rate levels and the metabolite levels in the sweat EVs isolated from healthy participants (N = 9; three women and six men) during exercise. *p < 0.05 is significant indicated as bold numbers.

Variable	Correlation with resting respiration rate	*P.* Value
Pyroglutamate	−0.35	0.359
Creatine	−0.17	0.68
Creatinine	−0.02	0.98
Glycine	−0.17	0.65
Alanine	−0.33	0.38
Arginine	0.18	0.64
Carnitine	0.07	0.88
Glutamate	−0.721	0.028 *
Glutamine	0.26	0.49
Lactate	−0.63	0.07
Proline	−0.12	0.78
Serine	−0.48	0.19
Threonine	−0.15	0.70
Tyrosine	0.1	0.81
Myristate	−0.77	0.02*
Palmitate	−0.72	0.03*

Overall, our results suggest that metabolites present in sweat EVs appear to correlate with cardiovascular system functions, however, given the limited number of participants, further validations are needed.

## Discussion

In this work, with the aid of clinically approved patches, we describe the metabolic profiling of sweat EVs isolated from healthy participants during exercise tests and following a recovery period, referred to as the recovery condition. Comparison of metabolite abundances between the two conditions reveals distinct metabolic patterns of sweat EVs in response to exercise. Furthermore, the correlation of metabolite levels in sweat EVs with blood glucose, BMI, and cardiovascular system functions suggests that metabolites can be used as potential biomarkers to non-invasively assess individualized sport performance in healthy participants.

Recently, we were the first to report a novel methodology allowing the enrichment, isolation, and characterization of sweat EVs from clinically approved patches, following heat exposure (Rahat et al., 2023). In this report, by performing a targeted metabolomics analysis, we have identified 24 metabolites. However, in the current report, and using the same patches and similar protocol for extraction of EVs, we were able to identify only sixteen metabolites.

The majority of the identified metabolites are similar to those previously described in Rahat et al., 2023 (Rahat et al., 2023), with few exceptions, such as the presence of metabolites involved in creatine metabolism pathway and the absence of metabolites belonging to TCA cycle pathway. This difference between the two studies indicates that heat exposure or exercise may influence the metabolic composition of sweat EVs, which may represent a powerful strategy to evaluate other stressors and expand the work to larger scale clinical studies.

Our current data identified lactate as one of the most abundant metabolites in sweat EVs in healthy participants during the exercise test and in the subsequent period of recovery. A significant upregulation of the lactate in sweat EVs during exercise was observed compared to its level in sweat EVs obtained after the recovery period. Furthermore, we found that lactate abundance in sweat EVs during exercise was positively associated with the mean resting blood pressure levels and negatively correlated with respiration rate levels. These results are in line with the literature demonstrating that a lactate shuttle is crucial for regulating the metabolic signaling associated with cardiovascular functions (Brooks, 2021; Li et al., 2022). In this phenomenon, lactate production is increased during exercise to fulfill the oxygen and ATP requirements of the cells and can be detected in the sweat (Harmer et al., 2008). Consequently, numerous studies propose lactate as a characteristic metabolite for monitoring physical activities (Durand et al., 2021; Seki et al., 2021; Torres-Torrelo et al., 2021; Okawara et al., 2022), and recently, wearable devices have been developed to monitor lactate (Garcia et al., 2016; Luo et al., 2018; Xuan et al., 2021; Kim et al., 2022). Considering our current data and the literature, we suggest that, once our preliminary observations are confirmed using a larger number of participants, monitoring lactate abundance in sweat EVs may be considered as a groundbreaking strategy to assess individualized sport performance during training in reference to the recovery state, namely, for athletes.

This study demonstrates that amino acids are significantly increased during exercise compared to recovery. Our data agree with previous investigations showing that during acute exercise, several amino acids such as alanine, glutamine, glutamate and the branched-chain amino acids are crucial for ensuring ATP production in muscle cells (Egan and Zierath, 2013; Hargreaves and Spriet, 2020; Smith et al., 2023). Furthermore, among these amino acids, glutamate shows a significant positive correlation with the mean resting blood glucose and negative association with the respiration rate levels in sweat EVs isolated during exercise. This pattern of association is similar to that observed with lactate.

Previous studies have revealed that pyroglutamate levels are biomarker for the diagnosis of heart failure (van der Pol et al., 2017; van der Pol et al., 2018; Bachhawat et al., 2020). Our data demonstrate that pyroglutamate is significantly upregulated in sweat EVs isolated during exercise test when comparing to its level in sweat EVs isolated at recovery. Moreover, a significant correlation is observed with pyroglutamate levels with heart rate at resting. Considering these studies and our data, we may speculate that pyroglutamate levels in sweat EVs can be used as an indirect indicator of cardiovascular system functions.

Our results reveal a decreased trend of both myristate and palmitate levels in sweat EVs isolated from healthy individuals during exercise compared with recovery periods. During exercise, fatty acids metabolism represents the main source of energy to supply the skeletal muscles (Hargreaves and Spriet, 2020), which may explain their decreased levels. However, depending on the duration of exercise, acute or chronic, the concentration of fatty acids in blood and adipose tissues could be influenced differently as previously reported (Nikolaidis and Mougios, 2004; Mika et al., 2019; Esmaili et al., 2023). Considering these reports and our data, we may propose that once our preliminary observation are confirmed using a larger number of participants, monitoring fatty acids in sweat EVs may be considered as a groundbreaking to gain insight for the function of skeletal muscles during training and recovery.

In our current study, the intensity of the two metabolites is associated negatively with the resting heart rate, the mean blood pressure levels including systolic and low frequency. In line with our observation, two epidemiological studies were conducted, one in Africa and one in China, to test the association between the concentration of fatty acids in blood serum with resting blood pressure levels. Findings from those studies highlighted that fatty acids were negatively associated with blood pressure (Yang et al., 2016; Zec et al., 2019). These data suggest protective effects of fatty acids against the risk of cardiac death via reducing resting heart rate ((Hidayat et al., 2018) and cytosolic and diastolic blood pressure in human (Mozaffarian and Wu, 2011). This was also underlined in animal study, in which a reduction of myocytes contraction and resting heart rate were observed following fatty acids exposure (Kang, 2012).

Given that those reports were performed to evaluate the fatty acids concentration in serum and no prior evidence showing the correlation between fatty acids in sweat or sweat EVs with cardiovascular system functions, our strategy may offer a novel non-invasive to test such association, However, further validation are required to draw conclusion. ion.

Overall, to our knowledge, our study is the first to show a distinct metabolic signature of sweat EVs enriched in sweat patches that have been isolated from healthy participants during exercise and recovery. The association of the identified metabolites with health parameters such as blood glucose, heart rate, and blood pressure may be useful for assessing and monitoring abnormal health related manifestations, namely, those associated with cardiovascular system functions, through a non-invasive approach.

### Study strengths and limitations

The strengths of the current study are the methodology allowing the enrichments and isolation of sweat EVs non-invasively during sport tests, the identification of metabolic composition in sweat EVs that represent potential biomarkers, and the proof-of-concept of a plausible correlation between the level of metabolite in sweat EVs with different physiological health parameters.

One notable limitation of the present study is the population size owing to the limited number of participants during exercise and at resting condition. Owing to this, and the high variation of metabolite concentrations in sweat EVs between individuals, the statistical power is small, hence, it becomes challenging to distinguish whether the alteration of metabolite levels derives from the exercise. Given these facts, performing extensive studies using large number of participants is therefore required to, first assess the metabolite levels in sweat EVs with regards to dissimilar conditions such as heat, glucose intervention, or others. Additionally, to investigate the association between exercise and the different set of physiological parameters. In the case of feasible correlation, subsequently, the metabolite can be used as possible biomarkers to predict any risk factors linked to heart function and cardiac performance during exercise. Additionally, in the current study, the only normalization approach used is the particle number/mL, although, more qualified methodologies are desired to better investigate the metabolic pattern of sweat before, during and after exercise and find the best normalization protocol.

When the obstacles mentioned above are clarified, we believe that the findings from this report may herald a strategy towards advancing individualized solutions for evaluating and surveilling health parameters and wellbeing in healthy and diseased individuals.

## Conclusion

Overall, the current study proposes a non-invasive approach to collect and isolate EVs from sweat with the aid of clinically approved patches worn during exercise. A targeted metabolomics analysis reveals the presence of a distinct composition of metabolites in sweat EVs. Furthermore, metabolite concentrations, mainly myristate, are correlated with a variety of physiological parameters, including blood glucose, heart rate, blood pressure, and respiration rate levels. Owing to the restricted number of participants and the high variation between individuals, the authors suggest expanding the study to large-scale cohorts to further confirm these observations.

In summary, we believe that sweat EVs, enriched using our methodology, are valuable tools to assess physiological actions of the human body during exercise and the methodology may be extended for a diverse range of clinical studies.

## Data Availability

The original contributions presented in the study are included in the article/Supplementary Material, further inquiries can be directed to the corresponding author.
