# Supplementary material for: Metabolic patterns of sweat-extracellular vesicles during exercise and recovery states using clinical grade patches
Source: Front Physiol. 2023 Dec 7;14:1295852. doi: 10.3389/fphys.2023.1295852 (PMC10748597; doi:10.3389/fphys.2023.1295852)
Supplement: Supplementary file 1 [file DataSheet1.PDF]

Supplementary Figure 1

A

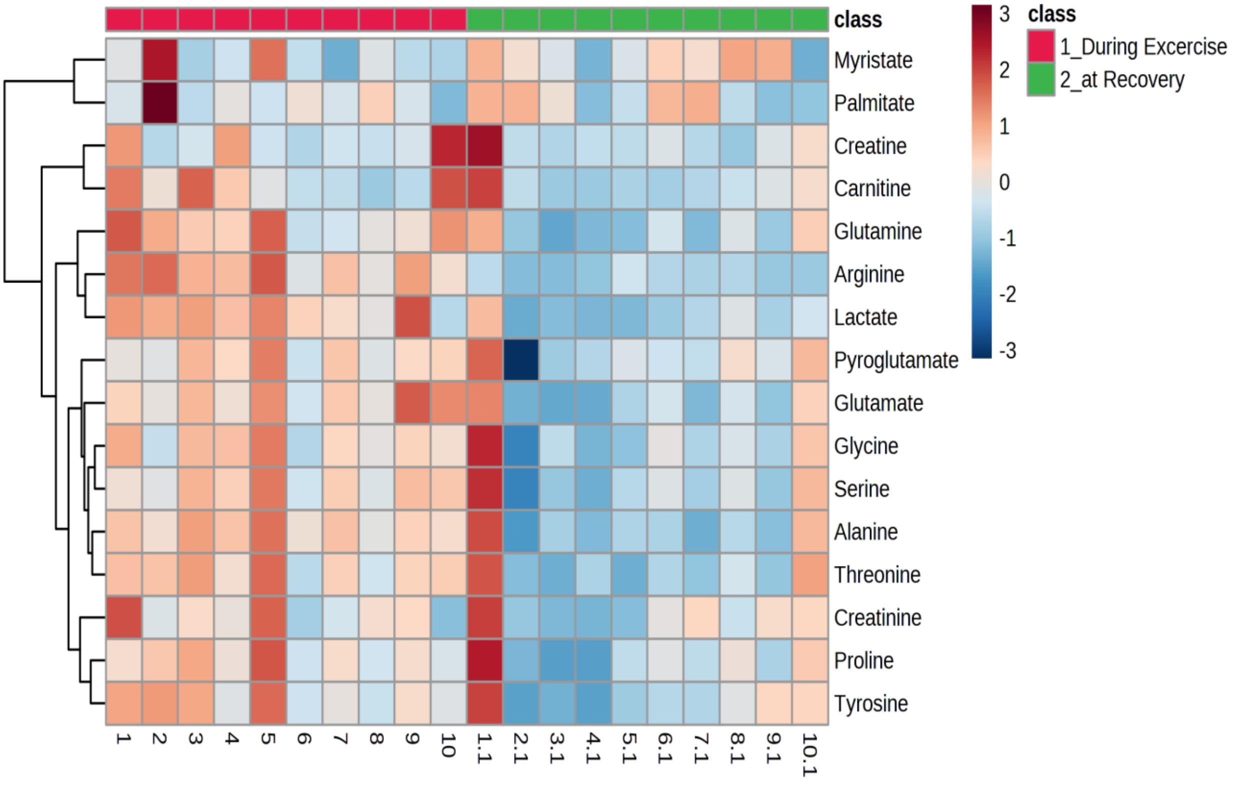

B

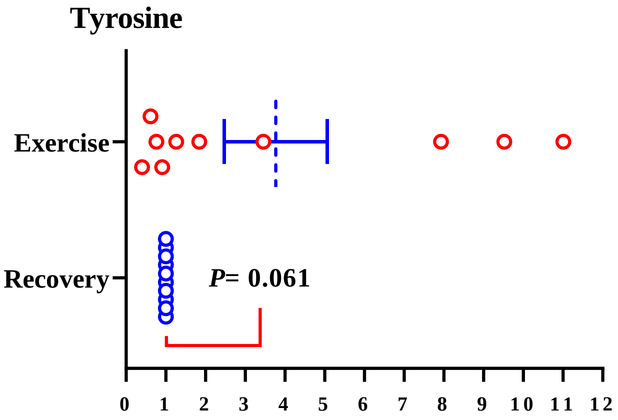

C

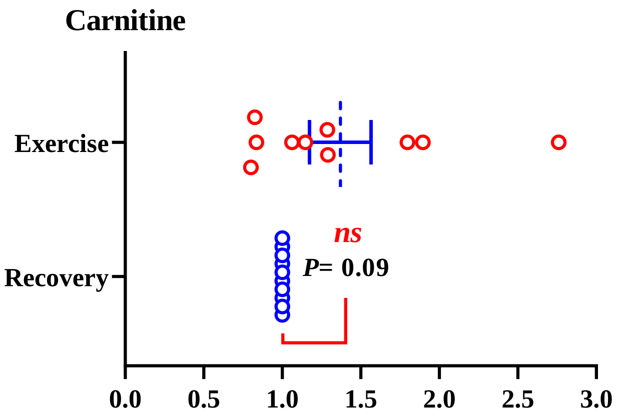

Supplementary Figure 2

A

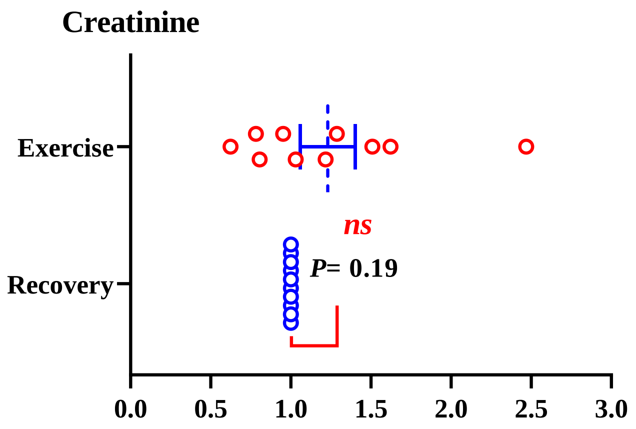

B

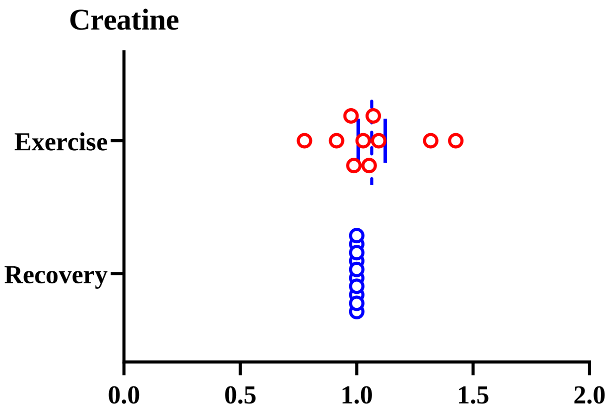

C

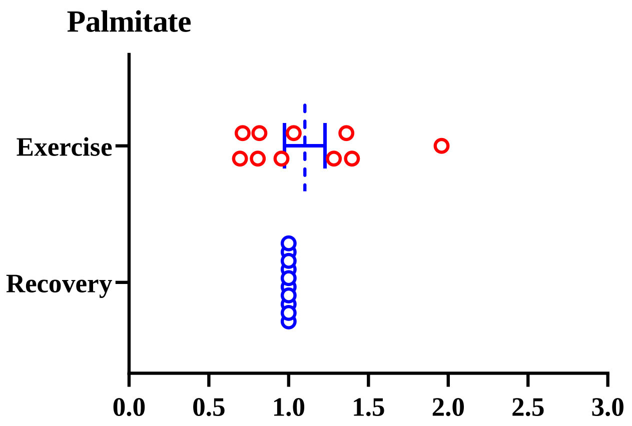

D

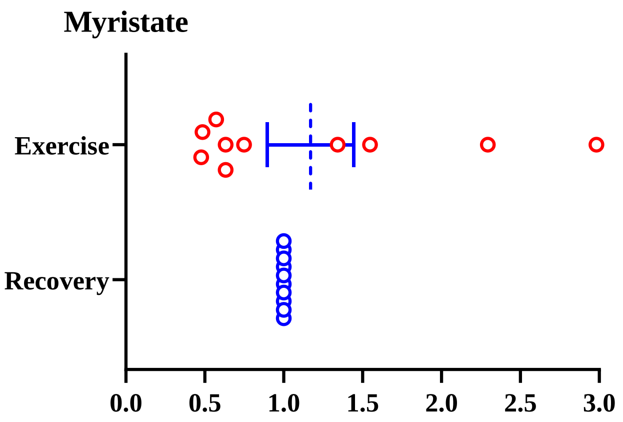

Supplementary Table 1

	Sweat EVs concentration (particles/ml)	
**Number**	**Exercise test**	**Recovery condition**
**1**	5.75×10^{10}	1.07×10^{10}
**2**	4.83×10^{10}	1.76×10^{10}
**3**	6.75×10^{10}	3.60×10^{10}
**4**	7.86×10^{09}	6.18×10^{10}
**5**	1.53×10^{10}	2.98×10^{10}
**6**	1.94×10^{10}	4.32×10^{10}
**7**	6.96×10^{09}	8.34×10^{09}
**8**	8.91×10^{07}	7.23×10^{10}
**9**	1.7×10^{07}	7.32×10^{08}
**10**	2.18×10^{08}	1.54×10^{10}

Supplementary Table 2

	Sweat EVs- Exercise										Sweat EVs - Recovery									
	1	2	3	4	5	6	7	8	9	10	1	2	3	4	5	6	7	8	9	10
**Pyroglutamate**	11744707	10550144	32545148	18762192	70177907	7184654	26066827	9803759	17777243	20512578	94900906	252039	3945082	5218488	9386153	7477293	6539376	15821474	9027887	31330550
**Creatine**	32343711	23602916	25079601	31947814	24736614	23432418	24902468	24461863	25297175	39364091	41664873	24175075	23397466	24243463	24042622	25626497	23631466	22347698	25581666	27612972
**Creatinine**	5754778	2963129	3472461	3192184	5387770	2408470	2859313	3349613	3514689	2209269	6036898	2301318	2140935	2114176	2182492	3077295	3545448	2751084	3408546	3536921
**Glycine**	2627641	911743	2328506	2177148	3858645	787609	1735870	1239817	1801894	1500363	7185563	297294	864801	507303	587192	1233955	758614	1085510	723872	2078378
**Alanine**	6006191	4180644	8397841	6050854	11851349	4064586	6127711	3527453	5199348	4420756	15308299	1097903	2044169	1562791	2205132	2157618	1364807	2358758	1648522	6651219
**Arginine**	16519840	18216240	9110122	8214468	21666678	3628095	7703263	4032949	11340531	4953883	2439451	1434676	1438125	1613602	2995607	2233259	2052917	2240253	1715581	1765120
**Carnitine**	7298674	4367629	8036326	5214574	3988586	3445647	3414101	2909585	3295725	8615370	9113092	3391491	2911286	2901383	3090950	3000570	3211617	3520090	3939534	4542724
**Glutamate**	1361584	949751	1851199	1078789	2693384	732989	1530154	940698	4064046	2790283	2931781	329023	294819	306102	527712	740924	355814	764277	407355	1375002
**Glutamine**	4581293	2695456	2121483	1990562	4345269	1117935	1216928	1474072	1617957	3196872	2634887	808428	606463	704174	735720	1244241	715526	1328762	839341	2049806
**Lactate**	250981564	212097309	236143220	171304807	298948802	142004356	120170803	91057031	455021076	57936493	178985071	30638564	36990830	34712339	35650095	44199513	55596658	84863822	50140256	74234921
**Lysine**	2691903	1600547	1267237	636516	1719059	876368	745596	566722	676647	634885	**NF**	**NF**	**NF**	**NF**	**NF**	**NF**	**NF**	**NF**	**NF**	**NF**
**Proline**	13967940	18587953	24733638	12935727	44537480	9166314	14496666	9509485	14307171	10285333	68520428	5016156	3958841	3985750	8324215	11088883	8219887	12781998	6964871	17960534
**Serine**	2692168	2156176	5216195	3707900	9534455	1743139	3912252	2020201	4673249	4235405	19048970	383132	963236	678158	1331064	2047779	1102681	2078069	948649	4913656
**Threonine**	2680784	2561409	3612566	1857141	5253744	1057404	2280759	1257505	2190936	2361827	6153122	713694	608396	955651	618541	983781	775438	1296892	787149	3453565
**Tyrosine**	5591958	6275929	5577809	1987309	9756429	1595929	2228188	1557428	2871951	2019512	13788580	569929	703629	575205	1024420	1262750	1206608	2041244	3167368	3261265
**Myristate**	6873722	23665273	4895403	6063231	15108541	5688490	3797546	6744280	5468657	5119292	10873306	7932918	6540539	3918335	6584059	8998198	7965456	11792590	11245470	3814024
**Palmitate**	304917635	835975356	274457397	322993208	291389434	341323676	302634016	377636576	301911438	227913980	428282763	426570049	339721574	231239340	282440613	417508300	434896663	277139435	235129627	238609724

Supplementary Table 3

Variable	Correlation with resting systolic blood pressure	*P.* Value
**Pyroglutamate**	0.01	0.98
**Creatine**	0.26	0.5
**Creatinine**	-0.34	0.37
**Glycine**	-0.19	0.62
**Alanine**	0.017	0.97
**Arginine**	-0.37	0.32
**Carnitine**	-0.15	0.69
**Glutamate**	0.47	0.17
**Glutamine**	-0.51	0.16
**Lactate**	0.42	0.27
**Proline**	-0.23	0.56
**Serine**	0.07	0.85
**Threonine**	-0.37	0.37
**Tyrosine**	-0.32	0.40
**Myristate**	-0.77	**0.01 ***
**Palmitate**	-0.23	0.54

Supplementary Table 4

Variable	Correlation with resting diastolic blood pressure	*P.* Value
**Pyroglutamate**	0.31	0.42
**Creatine**	0.36	0.34
**Creatinine**	-0.11	0.78
**Glycine**	-0.14	0.71
**Alanine**	0.16	0.68
**Arginine**	-0.14	0.72
**Carnitine**	-0.14	0.71
**Glutamate**	0.49	0.18
**Glutamine**	-0.39	0.29
**Lactate**	0.29	0.44
**Proline**	0.26	0.49
**Serine**	0.35	0.36
**Threonine**	-0.17	0.67
**Tyrosine**	-0.03	0.95
**Myristate**	-0.51	0.16
**Palmitate**	-0.2	0.61

Supplementary Table 5

Variable	Correlation with resting low frequency systolic blood pressure	*P.* Value
**Pyroglutamate**	0.28	0.463
**Creatine**	0.167	0.678
**Creatinine**	-0.233	0.552
**Glycine**	-0.001	0.997
**Alanine**	0.15	0.708
**Arginine**	-0.30	0.42
**Carnitine**	-0.13	0.74
**Glutamate**	0.44	0.24
**Glutamine**	-0.48	0.24
**Lactate**	0.29	0.44
**Proline**	0.07	0.88
**Serine**	0.33	0.39
**Threonine**	0.12	0.75
**Tyrosine**	-0.38	0.31
**Myristate**	-0.83	**0.008 ****
**Palmitate**	-0.4	0.29
